# Zinc oxide/copper ferrite ferrofluids derived from natural resources for antifungal applications

**DOI:** 10.1186/s11671-026-04816-y

**Published:** 2026-07-27

**Authors:** Febriana Rahmawati, Yap Wing Fen, ST. Ulfawanti Intan Subadra, Nurul Hidayat, Arif Hidayat, Budi Purnama, Munasir Munasir, Tahta Amrillah, Ahmad Taufiq

**Affiliations:** 1 Department of Physics, Faculty of Mathematics and Natural Sciences, Universitas Negeri Malang, Malang, Indonesia; 2 Department of Physics, Faculty of Sciences, Universiti Putra Malaysia, Seri Kembangan, Malaysia; 3 Department of Physics, Faculty of Mathematics and Natural Sciences, Universitas Sebelas Maret, Surakarta, Indonesia; 4 Department of Physics, Faculty of Mathematics and Natural Sciences, Universitas Negeri Surabaya, Surabaya, Indonesia; 5 Department of Nanotechnology Engineering, Faculty of Advanced Technology and Multidiscipline, Universitas Airlangga, Surabaya, Indonesia

**Keywords:** Zinc oxide/copper ferrite ferrofluids, Soursop leaf extract, Coconut oil, Superparamagnetic, Antifungal activity, *Aspergillusflavus*

## Abstract

One of the serious global health issues is related to toxigenic fungi, especially *Aspergillus flavus*. To address this issue, it is crucial to develop eco-friendly and effective antifungal agents. Therefore, we develop zinc oxide/copper ferrite–soursop leaf extract nanocomposites as novel antifungal agents using a combination of sol–gel, coprecipitation, and mixing methods. Interestingly, the nanocomposites were modified into ferrofluids with varying mass of copper ferrite–soursop leaf extract to increase their stability and antifungal performance. Furthermore, this work employed natural resources such as iron sand, soursop leaf, and coconut oil as the main eco-friendly precursors. The results of X-ray diffractometry showed that the nanocomposites formed two crystal phases, namely zinc oxide and copper ferrite with hexagonal wurtzite and inverse cubic spinel structures, respectively. Meanwhile, the presence of soursop leaf extract as a surfactant in ferrofluids was indicated by several main functional groups, such as C–H, O–H, and C=O. The morphology of the zinc oxide/copper ferrite–soursop leaf extract nanocomposites tended to be more uniform, with a size that decreased from 52.44 nm to 22.68 nm. The prepared ferrofluids were tested using the well diffusion method with 3 replicates, showing the antifungal activity against *Aspergillus flavus* with inhibition zone diameters ranging from 7.60 mm to 8.90 mm. This increase occurred because the particle size of nanocomposites was smaller, causing them to easily penetrate the fungal cell membrane. In addition, the increase in the inhibition zone diameter can be attributed to the presence of O–H functional groups and bioactive compounds, originating from increasing leaf extract composition. Furthermore, the data analysis confirmed that the increase in the inhibition zone diameter was not due to random variation, but rather the effectiveness of the ferrofluid system, as indicated by a statistically significant result (*p* < 0.05). The presence of oleic acid and dimethyl sulfoxide in the ferrofluids also contributed to increasing the stability of the nanocomposites and membrane penetration so that the ferrofluids enter the fungal cells. Furthermore, the synergistic effect between zinc oxide and copper ferrite also produced reactive oxygen species and interactions with fungal cells. These findings suggest that the prepared zinc oxide/copper ferrite–soursop leaf extract ferrofluids have potential as novel antifungal agents, especially against *Aspergillus flavus*.

## Introduction

In various industries, especially in the food and agriculture sectors, the presence of resistant fungi has become a serious issue, garnering significant scientific and industrial attention [1–3]. Such fungal contamination can attack crops from the pre-harvest to post-harvest stages, causing global yield reductions of up to 30% annually [4]. Additionally, in the food sector, it is reported that approximately 31% of the 133 billion pounds of food produced is lost or wasted [5]. This spoilage is generally driven by fungal contamination, which releases potent mycotoxins [6], posing significant threats to human health and food security. One of the fungi that plays a significant role is *Aspergillus flavus* (*A. flavus*), which is capable of producing aflatoxin [7, 8]. It becomes the most toxic type of mycotoxin that can impair liver and kidney function and even induce cancer [9–12]. This fungus is commonly found in soil with moist conditions [13, 14] and is also frequently distributed throughout the food chain, including in hay, cereals, and animal feed [4, 15]. Given its rapid growth capacity and detrimental impacts, developing strategies to inhibit the proliferation of *A. flavus* is crucial, highlighting the potential of nanomaterials as innovative antifungal agents.

Zinc oxide is one of the nanomaterials currently being widely explored as an agent to overcome fungal contamination due to its capability to generate the formation of free radicals that damage the cell membrane, triggering protein leakage and inhibiting fungal growth [16]. In addition, zinc oxide has excellent biocompatibility properties [17], is non-toxic, and does not cause allergies or irritation [18, 19], making it safe upon ingestion [20, 21]. Even in medicine, ZnO can be found as one of the ingredients that is widely used in several creams to treat skin infections caused by fungi [22, 23]. Previous research has shown that the incorporation of zinc oxide significantly reduces the number of *A. flavus* colonies in culture media [24]. However, zinc oxide has limitations due to its relatively wide band gap, resulting in slow electron–hole recombination, which impacts its antifungal performance [25, 26]. Therefore, this problem can be overcome by integrating with narrow band gap materials, such as copper ferrite [27]. Previous research reported that combining zinc oxide with copper ferrite resulted in a narrower band gap, which resulted in improved antimicrobial performance [28]. Apart from that, copper ferrite is very promising to be developed as an antifungal due to its biocompatibility [29], selective toxicity to living cells [30], and ability to interact with microbes, generating electrostatic forces that encourage the production of reactive oxygen species [31]. However, at the nanoscale, copper ferrite tends to agglomerate, which can reduce its interaction effectiveness with microbes [32]. Therefore, to overcome this issue, this study incorporated soursop leaf extract (*Annona muricata L.*), containing phytochemicals believed to prevent nanoparticle agglomeration [33]. Some of the photochemical compounds contained in soursop leaf extract are alkaloids, flavonoids, phenolics, and saponins [34]. According to previous research, some of these compounds, especially phenolics, can form stable complexes with metal ions that aim to coat nanoparticles to reduce agglomeration and form more stable particles [35]. Some of these components have hydroxyl functional groups, such as –OH, that interact and attach to the surface of nanoparticles. These functional groups can provide repulsive forces between particles, preventing them from sticking together and reducing agglomeration. Interestingly, the photochemical compounds contained in soursop leaf extract are also believed to contribute to the treatment of fungal infections due to their ability to enhance interactions with bacterial cells and their anti-inflammatory activity. Previous research successfully demonstrated that soursop leaf extract can inhibit the growth of *Candida albicans* [36, 37].

To date, various studies have been developed by incorporating zinc oxide with magnetic materials and/or plant extracts as antifungal agents, such as against *Candida albicans* [38]. However, the addition of pomegranate peel extract to the synthesis of zinc oxide also showed effective antifungal activity to inhibit the growth of several types of *Candida* fungus [39]. Previous research synthesized zinc oxide/magnetite using camel urine as an antifungal agent against *Aspergillus fumigatus*. However, no inhibition zone was obtained due to the low agglomeration and stability of the nanocomposite [40]. Meanwhile, material stability also plays an essential role in the antimicrobial agent activity [41].

Interestingly, to address this issue, this study also modified the nanocomposite form into a ferrofluid with the aim of forming a stable colloidal suspension [42]. This study formulated modified zinc oxide/copper ferrite–soursop leaf extract ferrofluids using a double surfactant system comprising oleic acid and dimethyl sulfoxide [43]. Oleic acid was selected due to its ability to maintain the dispersion of the nanocomposite [44, 45]. However, previous studies have shown that oleic acid alone is insufficient to maintain long-term filler stability [41]. Meanwhile, dimethyl sulfoxide has been shown to effectively maintain filler stability over extended periods [46, 47], can dissolve polar and nonpolar compounds while also playing a role in creating bioactive fractions for antimicrobial activity [48]. Furthermore, the addition of a dispersant is also required in ferrofluids; coconut oil was chosen as the dispersing medium due to its ability to disperse OA [49] to reduce nanoparticle agglomeration [50, 51], providing medical potential as an antimicrobial and immunostimulant agent [52]. So far, although various research reports have indicated that ferrofluids have been developed for antimicrobial applications [41, 43, 53], research reports related to the development of zinc oxide/copper ferrite–soursop leaf extract ferrofluids as antifungal agents against *A. flavus* are difficult to find.

This study aimed to produce zinc oxide/copper ferrite–soursop leaf extract ferrofluids as antifungal agents against *A. flavus*. Furthermore, this study also investigated the mass composition effect of copper ferrite–soursop leaf extract on the antifungal performance of ferrofluids using the well diffusion method. In addition, to verify the success of the synthesis and to understand the material properties, a series of characterizations in evaluating the crystal structure, functional groups, morphology, and magnetic properties of zinc oxide/copper ferrite–soursop leaf extract was also conducted. Interestingly, in addition to the use of soursop leaf extract, this study also used natural materials derived from iron sand and coconut oil as precursors for producing copper ferrite and dispersants in the ferrofluids. The use of these natural materials is very important to reduce production costs, offering an environmentally friendly, biocompatible, and sustainable alternative compared to the use of conventional chemicals.

## Materials and methods

### Materials

Iron sand was obtained from Sine beach, Tulungagung, Indonesia. Zinc acetate dihydrate (Zn(OOCCH_3_)_2_·2H_2_O), copper dichloride (CuCl_2_·2H_2_O), dimethyl sulfoxide (CH_3_)_2_SO, hydrochloric acid (HCl) 12 M, ammonium hydroxide (NH_4_OH) 6.5 M, and sodium hydroxide (NaOH) 3 M were purchased from *Merck* in analytical grade without further purification. The virgin coconut oil, soursop leaf, oleic acid, distilled water, deionized water, and Sabouraud dextrose agar (SDA) were also employed in this work.

### Synthesis of zinc oxide nanoparticles

The first step of the synthesis was conducted to prepare ZnO nanoparticles using the sol–gel method, following the steps described in our previous work [54, 55]. The first step was to dissolve 6.57 g of Zn(OOCCH_3_)_2_.2H_2_O in deionized water and then titrate with NaOH 3 M to pH 13, followed by stirring for 60 min at room temperature. The reaction result formed (Zn(OH)_2_), as shown in Eq. (1). Furthermore, (Zn(OH)_2_) was heated at 90 °C for 30 min to form ZnO, as shown in Eq. (2), followed by washing the sample with methanol until the pH was neutral. The precipitate obtained was then dried at 100 °C for 60 min.

1 $$ {\mathrm{Zn}}\left( {{\mathrm{OOCCH}}_{{3}} } \right)_{{2}} .{\mathrm{2H}}_{{2}} {\mathrm{O}} + {\mathrm{2NaOH}} \to {\mathrm{Zn}}\left( {{\mathrm{OH}}} \right)_{{2}} + {\mathrm{2CH}}_{{3}} {\mathrm{COONa}} + {\mathrm{H}}_{{2}} {\mathrm{O}} $$

2 $$ {\mathrm{Zn}}\left( {{\mathrm{OH}}} \right)_{{2}} \to {\mathrm{ZnO}} + {\mathrm{H}}_{{2}} {\mathrm{O}} $$

### Preparation of soursop leaf extract

The soursop leaf extract was prepared by the maceration method, using soursop leaves as the main precursor. First, the soursop leaves were thoroughly washed, cut into pieces, and then oven-dried for 1 h. Once dry, the leaves were ground and sieved to form a fine powder. This powder was then dissolved in deionized water at a ratio of 1:10 (g:mL) and stirred at 100 °C for 30 min. Finally, the final product was then filtered to produce the soursop leaf extract solution.

### Synthesis of copper ferrite–soursop leaf extract nanoparticles

In this study, the synthesis of the copper ferrite–soursop leaf extract ferrofluids started with the separation of iron sand to obtain magnetite (Fe_3_O_4_) powder. The 20 g of Fe_3_O_4_ was reacted with 58 mL of HCl 12 M while stirring at 720 rpm to produce Fe^2+^ and Fe^3+^ ions, as shown in Eq. (3). Next, the Fe^2+^ and Fe^3+^ ions from this reaction reacted with Cu^2+^ ions derived from CuCl_2_ 2H_2_O. In this process, the reaction was stirred for 10 min. Then, 3 mL of soursop leaf extract solution was added to the mixture and then titrated with 25 mL of NH_4_OH 6.5 M under stirring using a magnetic stirrer. In this process, subsequent alkalinization with NH₄OH 6.5 M resulted in the precipitation of mixed-metal hydroxides, as shown in Eq. (4). The reaction product was then washed until the pH reached 7.

3 $$ {\mathrm{Fe}}_{{3}} {\mathrm{O}}_{{4}} + {\mathrm{8HCl}} {\mathrm{FeCl}}_{{2}} + {\mathrm{2FeCl}}_{{3}} + {\mathrm{4H}}_{{2}} {\mathrm{O}} $$

4 $$ {\mathrm{Fe}}^{2 + } + {\mathrm{2Fe}}^{3 + } + {\mathrm{Cu}}^{2 + } + {\mathrm{8OH}}^{ - } \to {\mathrm{Fe}}\left( {{\mathrm{OH}}} \right)_{2} {\text{ + 2Fe}}\left( {{\mathrm{OH}}} \right)_{3} + {\mathrm{Cu}}\left( {{\mathrm{OH}}} \right)_{2} $$

At this stage, Fe(OH)_2_ was oxidized to Fe^3+^ and the hydroxide undergoes dehydration and a solid reaction to form copper ferrite oxide (CuFe₂O₄), as shown in Eq. (5).

5 $$ {\mathrm{Cu}}\left( {{\mathrm{OH}}} \right)_{{2}} + {\mathrm{2Fe}}\left( {{\mathrm{OH}}} \right)_{{{3} }} \to {\mathrm{CuFe}}_{{2}} {\mathrm{O}}_{{4}} + {\mathrm{4H}}_{{2}} {\mathrm{O}} $$

### Synthesis of zinc oxide/copper ferrite–soursop leaf extract ferrofluids

**Fig. 1:**
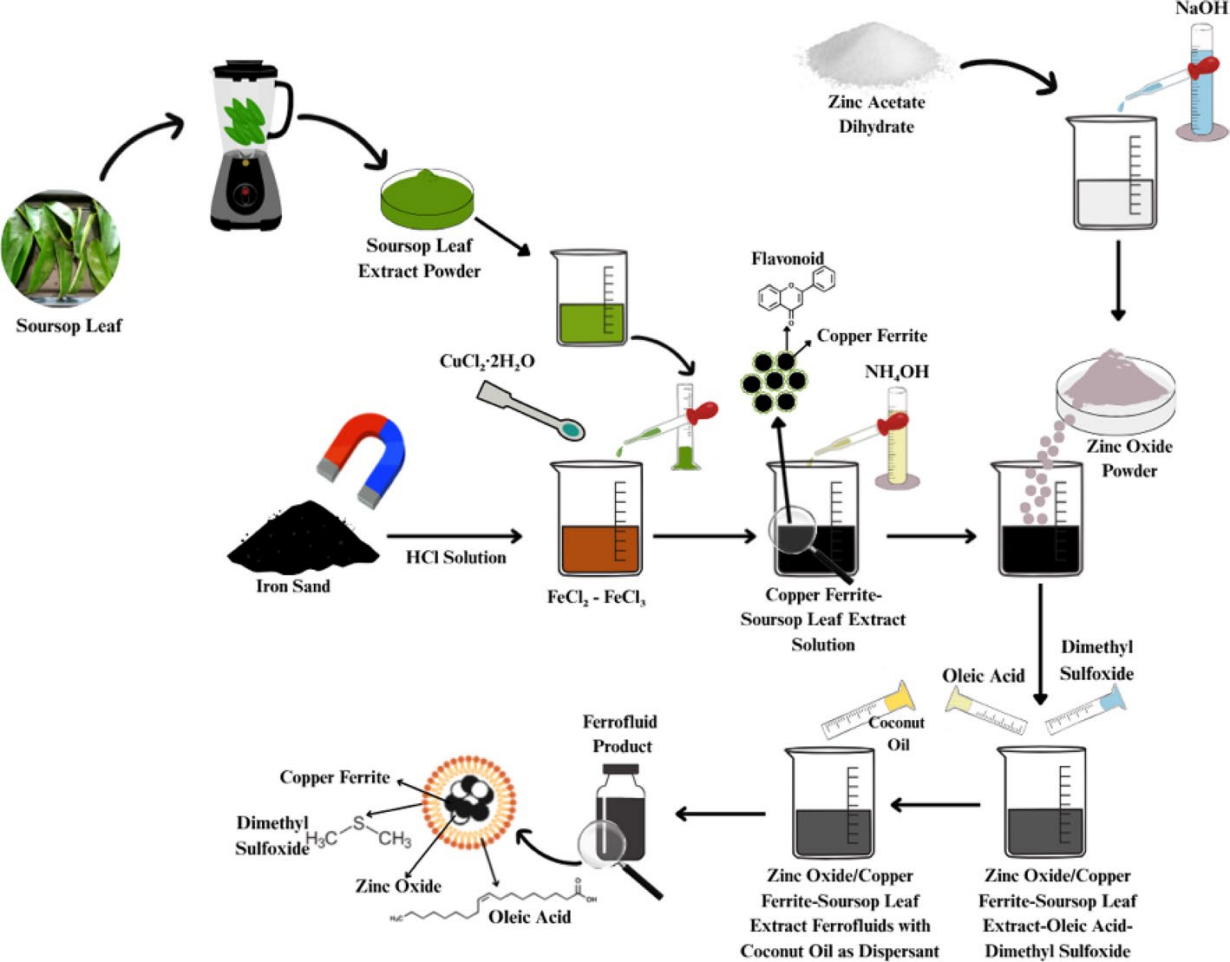
Synthesis procedure for the zinc oxide/copper ferrite–soursop leaf extract ferrofluids

The synthesis of zinc oxide/copper ferrite–soursop leaf extract ferrofluids started with the preparation of zinc oxide/copper ferrite–soursop leaf extract nanocomposites using a conventional mixing method. The copper ferrite–soursop leaf extract precipitate with varying masses (4, 5, 6, 7, and 8 g) was dissolved in 25 mL of deionized water and stirred at 720 rpm for 1 h. Next, 0.1 g of zinc oxide nanoparticles was added to the copper ferrite–soursop leaf extract solution, which was then stirred for 1 h using a magnetic stirrer. 1 g zinc oxide/copper ferrite–soursop leaf extract precipitate from each variation was dissolved in 2 mL of oleic acid (OA) and 2 mL dimethyl sulfoxide, under stirring at 720 rpm for 1 h. OA was the first surfactant to coat the surface of zinc oxide/copper ferrite–soursop leaf extract. OA, which contains carboxylate functional groups (–COOH) and hydrocarbon chains, interacted with the nanocomposites. The carboxylate functional groups bound to the positive ions in the nanocomposites and formed a protective layer, while the hydrocarbon chains provided steric hindrance, both of which prevented agglomeration and maintained the stability of the nanocomposites. Furthermore, dimethyl sulfoxide (DMSO), which has amphiphilic properties, facilitated the homogeneous distribution of the nanocomposites within the ferrofluid system. After that, 3 mL of coconut oil was added to the solution and stirred for 1 h at 90 °C. Each sample was coded NF1, NF2, NF3, NF4, and NF5. Some of the zinc oxide/copper ferrite–soursop leaf extract nanocomposites were then dried for characterization using X-ray diffraction (XRD), scanning electron microscopy–energy dispersive X-ray (SEM–EDX), and vibrating sample magnetometer (VSM). Meanwhile, the zinc oxide/copper ferrite–soursop leaf extract ferrofluids were characterized using transform infrared (FTIR) spectroscopy and evaluated for antifungal activity. The detailed stages of the ferrofluid synthesis are shown in Fig. 1.

### Antifungal activity testing of the zinc oxide/copper ferrite–soursop leaf extract ferrofluids

Antifungal activity testing of the zinc oxide/copper ferrite–soursop leaf extract ferrofluids against *A. flavus* was conducted using the well diffusion method. The first step was to prepare the fungal growth medium in the form of SDA. 25 g of SDA was dissolved in 1.000 mL of distilled water, followed by heating and stirring until the solution boiled and became clear. The SDA solution was then poured into a petri dish, sterilized, and left for 24 h. After the compaction process, *A. flavus* was slowly and evenly poured into the seed medium and placed in an incubator at room temperature for 24 h. Furthermore, a 6 mm hole was made in the Petri dish for each variation, and 3 replicates were carried out for each variation. A volume of 0.2 mL of ferrofluid was added to the well and then placed in an incubator at 27 °C for 24 h. Afterward, the inhibition zone around the hole or well, marked as the clear zone, was observed and measured. In this study, measurements were taken horizontally and vertically. Then, the inhibition zone data from three replicates were analyzed using a one-way analysis of variance (ANOVA), with differences considered significant for *p < 0.05.

### Characterization

XRD characterization was performed using a Panalytical X'Pert Pro device with Cu-Kα 1.540-Å to determine the crystal structures and phases formed in the nanocomposites. The morphology and constituent elements in the nanocomposites were characterized using an SEM–EDX FEI Inspect-S50. The nanocomposites were also characterized using VSM (a cryogen-free 3-T Quantum Design Physical Property Measurement System VersaLab) to determine their magnetic properties. Meanwhile, the zinc oxide/copper ferrite–soursop leaf extract ferrofluids were characterized using FTIR spectroscopy (Shimadzu IR Prestige-21) to analyze their functional groups. The ferrofluids were also evaluated for antifungal activity by measuring the inhibition zone diameter of *A. flavus* using the well diffusion method.

## Results

### Characterization

#### XRD analysis of zinc oxide/copper ferrite–soursop leaf extract nanocomposites

**Fig. 2:**
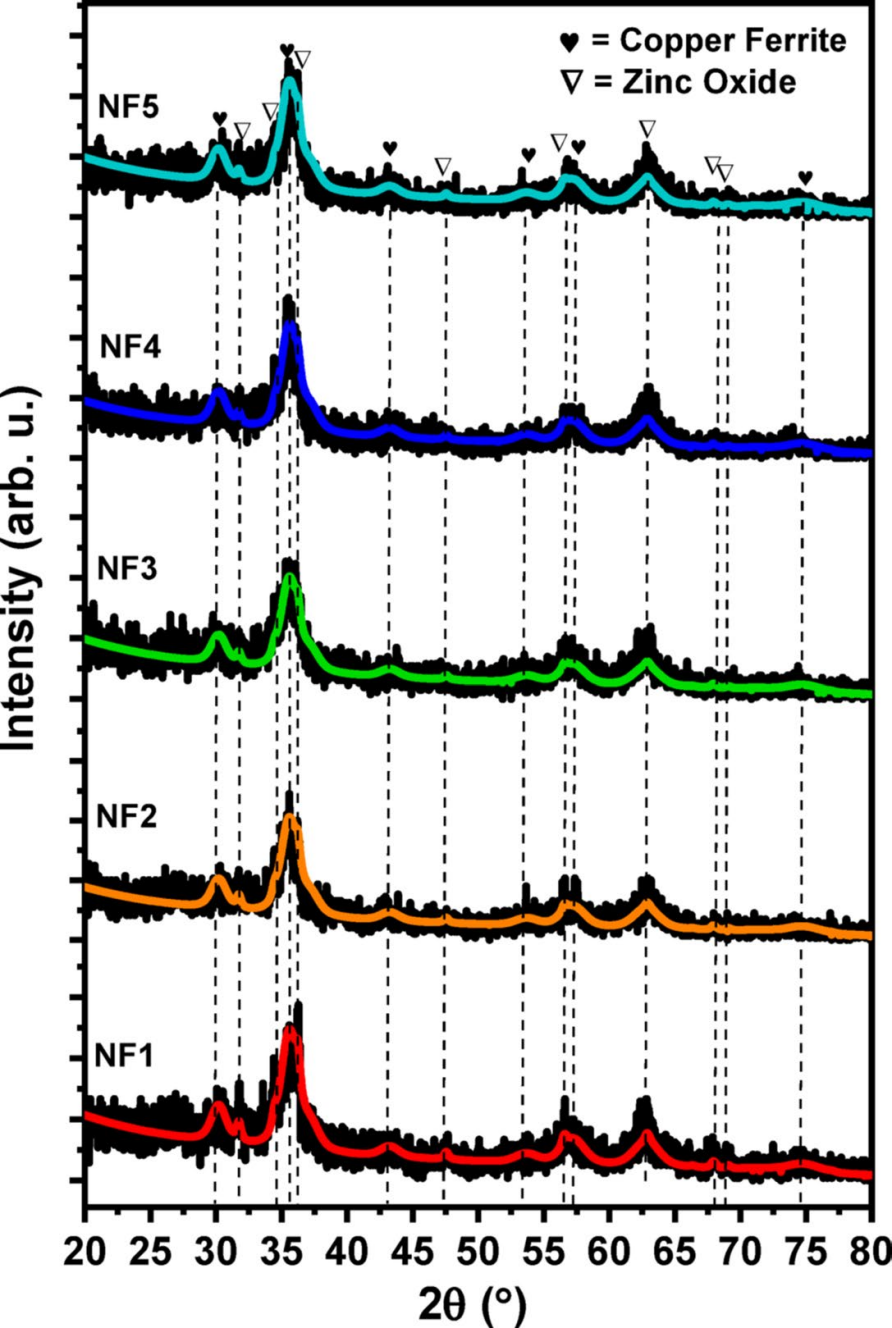
Diffraction patterns of the nanocomposites

The diffraction patterns of the zinc oxide/copper ferrite–soursop leaf extract nanocomposites with sample codes NF1, NF2, NF3, NF4, and NF5 are shown in Fig. 2. Qualitatively, there is a match between the diffraction patterns of the experimental results with the database of zinc oxide (AMCSD 0015176) and copper ferrite (AMCSD 0012012). The match between the experimental results shows that zinc oxide and copper ferrite have the same structure as the database in cubic and hexagonal structures. In this study, the diffraction peaks of zinc oxide were detected at 2θ = 31.8°, 34.5°, 36.2°, 47.6°, and 56.6°, 62.9°, 67.3°, and 69.0° correspond to hkl planes (010), (200), (110), (210), (110), (013), (112), and (120). These results are in line with the results of previous studies, which reported that zinc oxide peaks appeared at 2θ = 31.8°, 34.4°, and 36.3°, which correspond to hkl (001), (002), and (011), respectively, constructing a hexagonal wurtzite structure [54]. Meanwhile, copper ferrite peaks were detected at 2θ = 30.3°, 35.6°, 43.3°, 53.6°, and 57.2°, which correspond to hkl planes (220), (311), (400), (422), (511), and (533), respectively, constructing an inverse cubic spinel structure. The presence of two characteristic peaks from zinc oxide and copper ferrite confirms the successful formation of a binary composite from these two materials [56]. Interestingly, the absence of new peaks after the presence of the extract confirms that soursop leaf extract successfully acts as a stabilizing agent to reduce agglomeration [57].

Furthermore, as the mass of the copper ferrite–soursop leaf extract increased, there is no shift in either the copper ferrite or zinc oxide peaks. In addition, no lattice contraction was observed, as indicated by the unchanged lattice parameters: copper ferrite with *a* = *b* = *c* = 8.366 Å and zinc oxide with *a* = *b* = 3.254 Å, *c* = 5.205 Å. Interestingly, the peak intensity of copper ferrite increased slightly, though not significantly, with the addition of the copper ferrite–soursop leaf extract. This increase in diffraction peak intensity correlates with the higher percentage phase of the copper ferrite, as shown in Table 1, which increased from 51.61% to 61.12%. This increase was also accompanied by a decrease in the percentage phase of zinc oxide. These results confirm that the copper ferrite phase in the nanocomposites increases with the addition of more copper ferrite–soursop leaf extract. In addition, there was also a broadening of the peaks in the diffraction patterns, which can be associated with the FWHM value of each phase. The FWHM value is smaller, the crystallite size obtained is larger. As a result, the crystallite size of copper ferrite increases from 11.75 to 11.91 nm, and the crystallite size of zinc oxide increases from 29.79 to 29.84 nm.

**Table 1:** Results of X-ray diffraction analysis of the nanocomposites

Parameters	NF1	NF2	NF3	NF4	NF5
*Crystallite size (nm)*					
Copper ferrite	11.75	11.79	11.82	11.88	11.91
Zinc oxide	29.83	29.79	29.83	29.87	29.84
*Phase percentage (%)*					
Copper ferrite	51.61	55.99	58.50	61.31	61.12
Zinc oxide	48.39	44.01	41.50	39.69	38.88
*Lattice parameters (Å)*					
Copper ferrite–soursop leaf extract	8.366	8.366	8.366	8.366	8.366
Zinc oxide	3.254	3.254	3.254	3.254	3.254
	5.205	5.205	5.205	5.205	5.205
*Full width at half maximum (FWHM)*					
Copper ferrite	1.192	1.098	1.060	1.037	0.979
Zinc oxide	0.190	0.216	0.192	0.183	0.189

#### FTIR analysis of zinc oxide/copper ferrite–soursop leaf extract ferrofluids

**Fig. 3:**
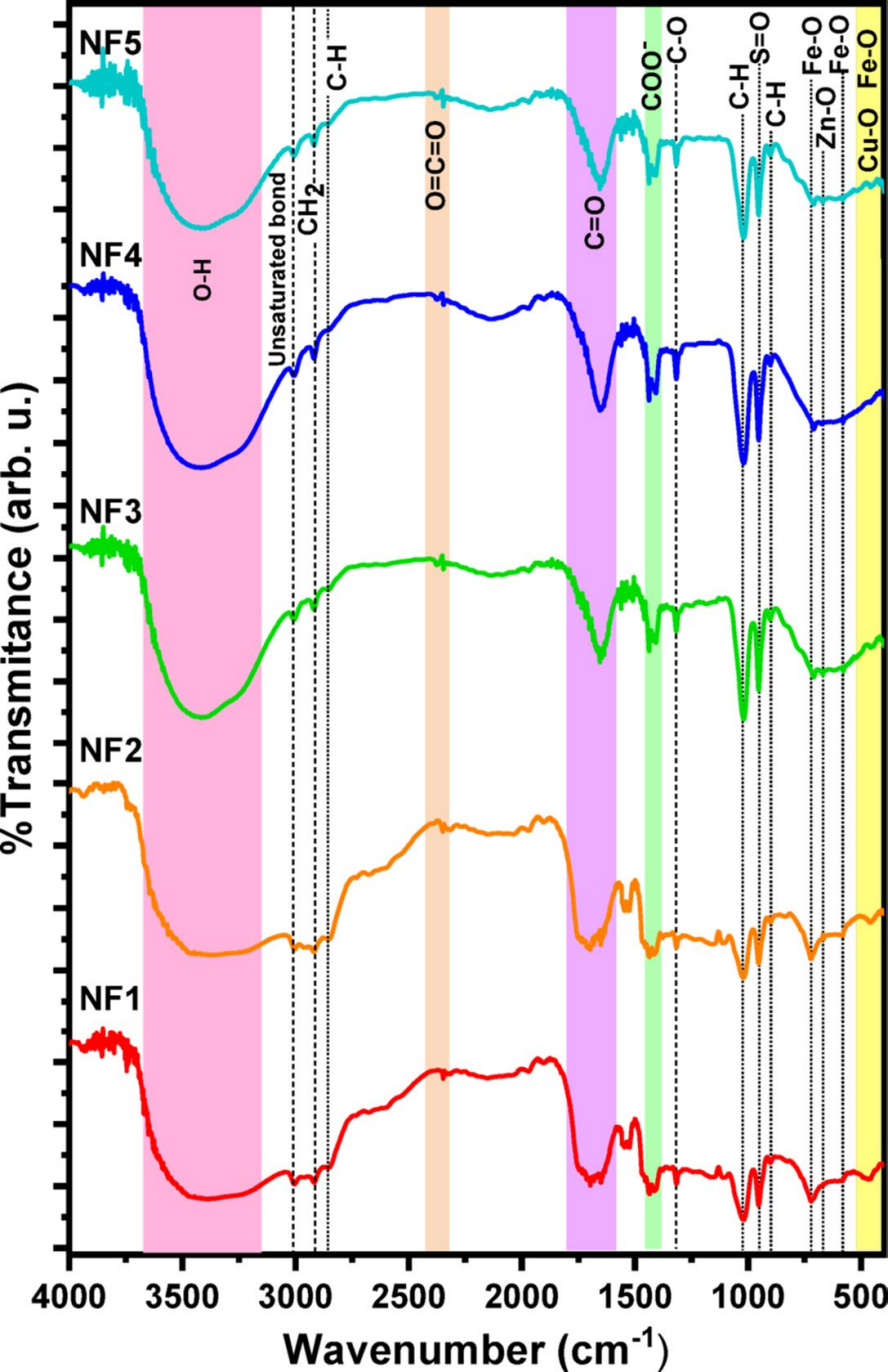
FTIR spectrum of the ferrofluids

The FTIR spectrum of zinc oxide/copper ferrite–soursop leaf extract ferrofluids is shown in Fig. 3. FTIR results confirmed the presence of octahedral Fe–O, Cu–O, tetrahedral Fe–O, and Zn–O. The presence of these groups indicates the presence of zinc oxide/copper ferrite-soursop leaf extract nanocomposites. In addition, C–H bonds appeared at 894 cm^−1^, 1018 cm^−1^, and 2858 cm^−1^, which indicated the presence of organic compounds in the ferrofluids. Furthermore, the presence of surfactants (OA and DMSO) and dispersants in the ferrofluids was detected from S=O, C–O, COO^−^ C=O, CH_2_, and O=C=O. In addition, the absorption bands observed at 3405 cm^−1^ and 3398 cm^−1^ indicated the presence of O–H vibrations.

#### SEM–EDX analysis of zinc oxide/copper ferrite–soursop leaf extract nanocomposites

**Fig. 4:**
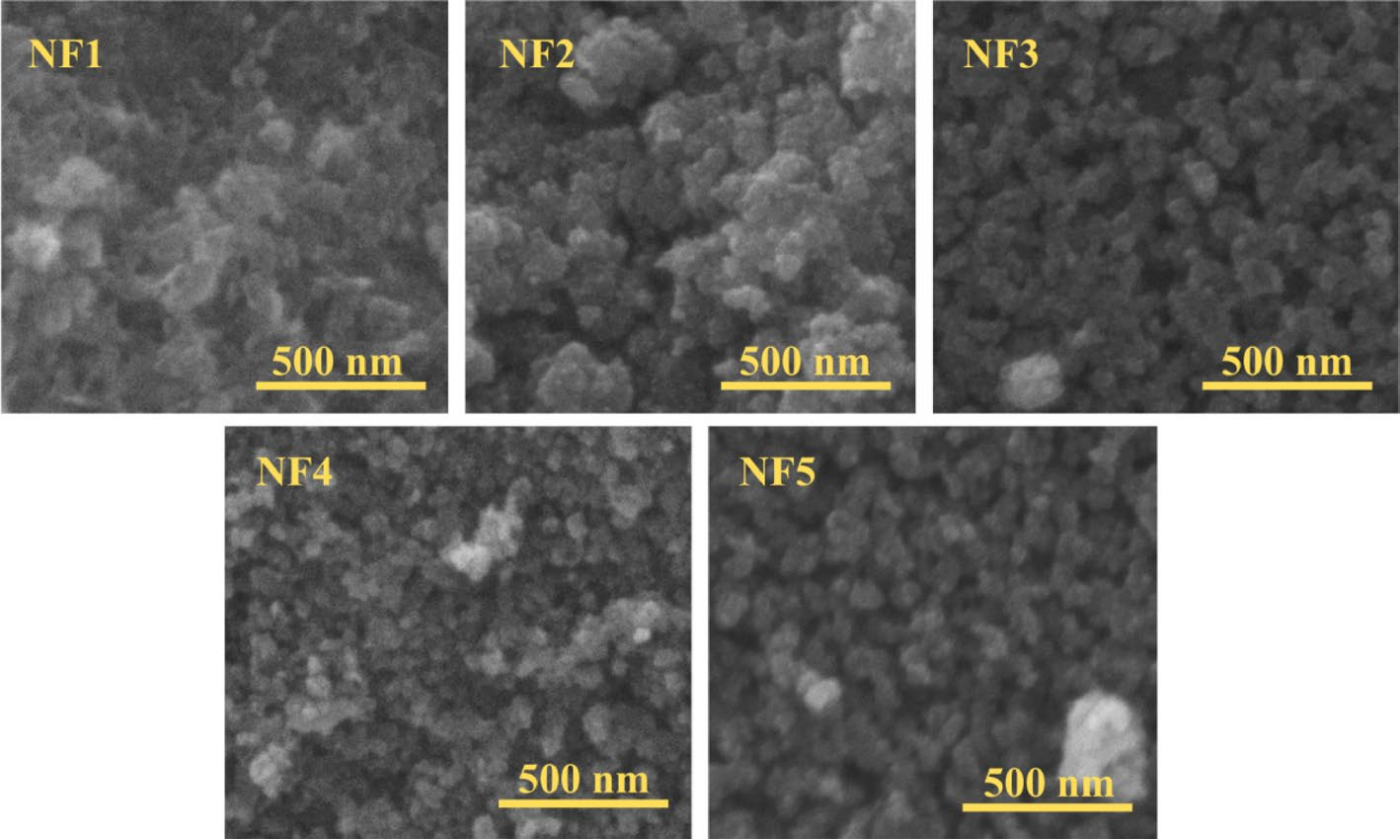
SEM images of the nanocomposites

**Fig. 5:**
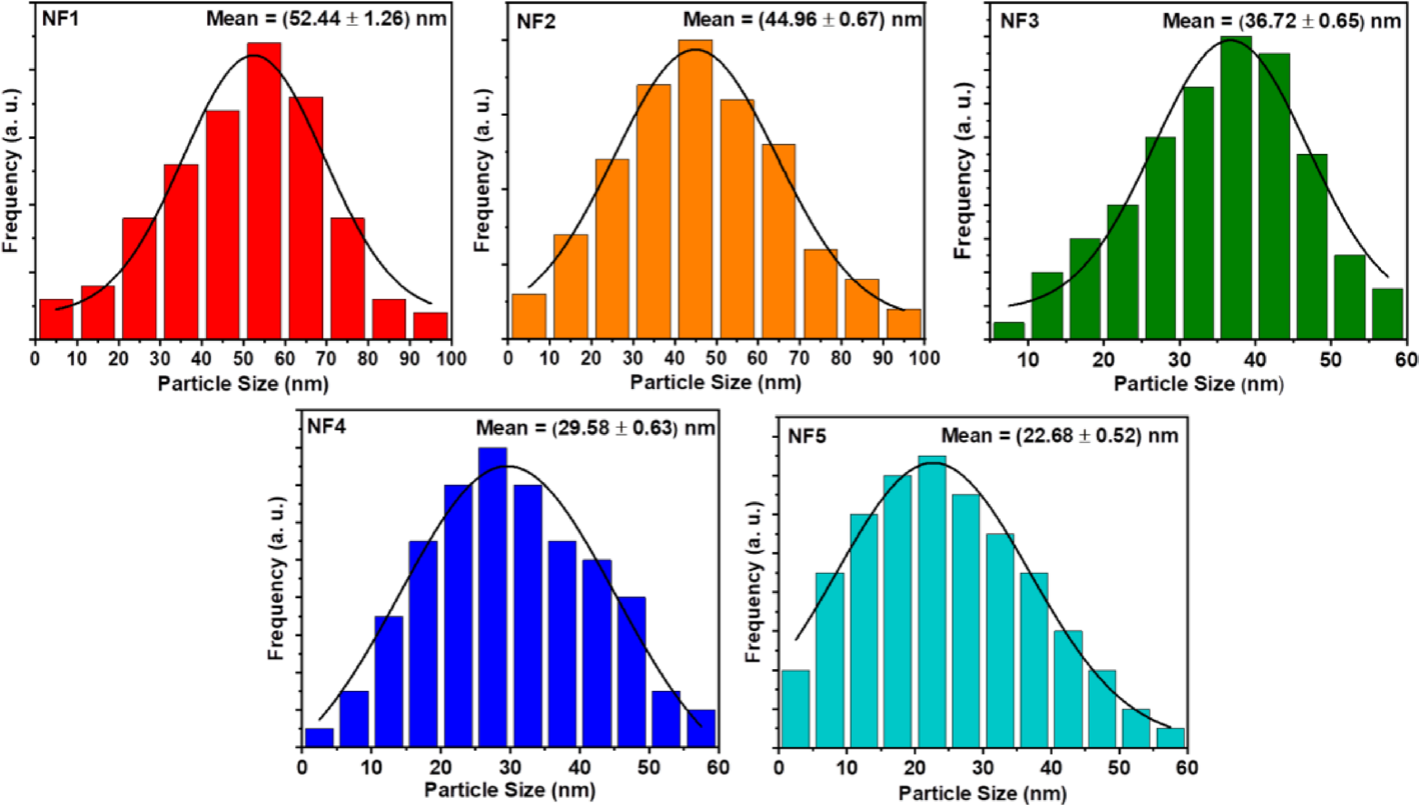
Particle size of the nanocomposites

**Fig. 6:**
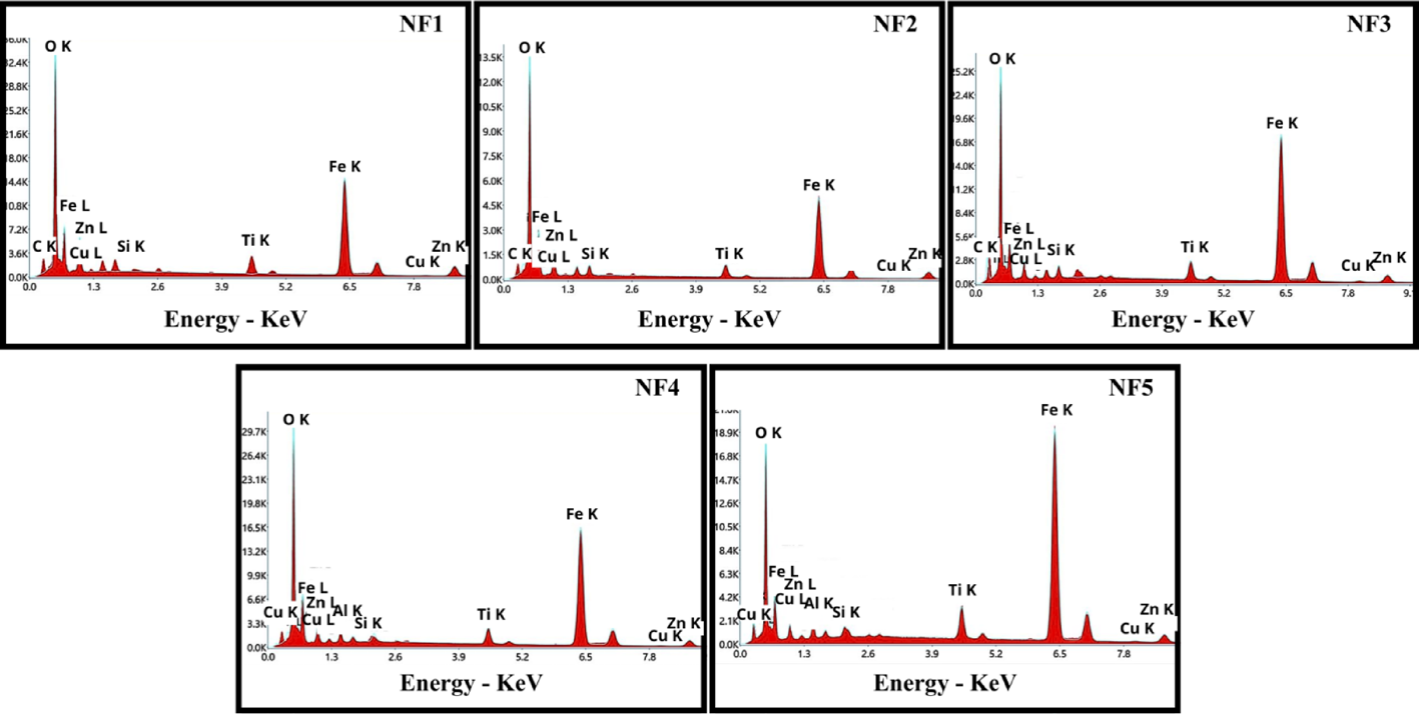
EDX spectra of the nanocomposites

Figure 4 shows the morphology of zinc oxide/copper ferrite–soursop leaf extract characterized by SEM. Qualitatively, the morphology of the nanocomposites exhibits dark and light regions corresponding to spherical particles. With the increasing amount of copper ferrite–soursop leaf extract, the morphology of the nanocomposites becomes more uniform in both shape and size, accompanied by reduced agglomeration. The particle sizes of zinc oxide/copper ferrite–soursop leaf extract, as shown in Fig. 5, are 52.44 ± 1.26 nm, 44.96 ± 0.67 nm, 36.72 ± 0.65 nm, 29.58 ± 0.63 nm, and 22.68 ± 0.52 nm for NF1, NF2, NF3, NF4, and NF5 samples, respectively. Based on these data, it is known that the particle size decreases with increasing mass of copper ferrite–soursop leaf extract. Furthermore, the EDX analysis of the nanocomposites presented in Fig. 6, shows the presence of oxygen (O) at an energy of around 0.5 keV, iron (Fe) at around 6.5 and 0.7 keV, copper (Cu) at around 0.9 keV, zinc (Zn) at around 1 keV, and carbon (C) is also detected in all samples at a low energy range of around 0.3. Furthermore, the content of aluminum (Al), silicon (Si), titanium (Ti), and chlorine (Cl) elements in small percentages was also detected at energies of around 1.5 keV, 1.7 keV, 0.6 keV, and 2.6 keV.

### Magnetic properties of zinc oxide/copper ferrite–soursop leaf extract nanocomposites

**Fig. 7:**
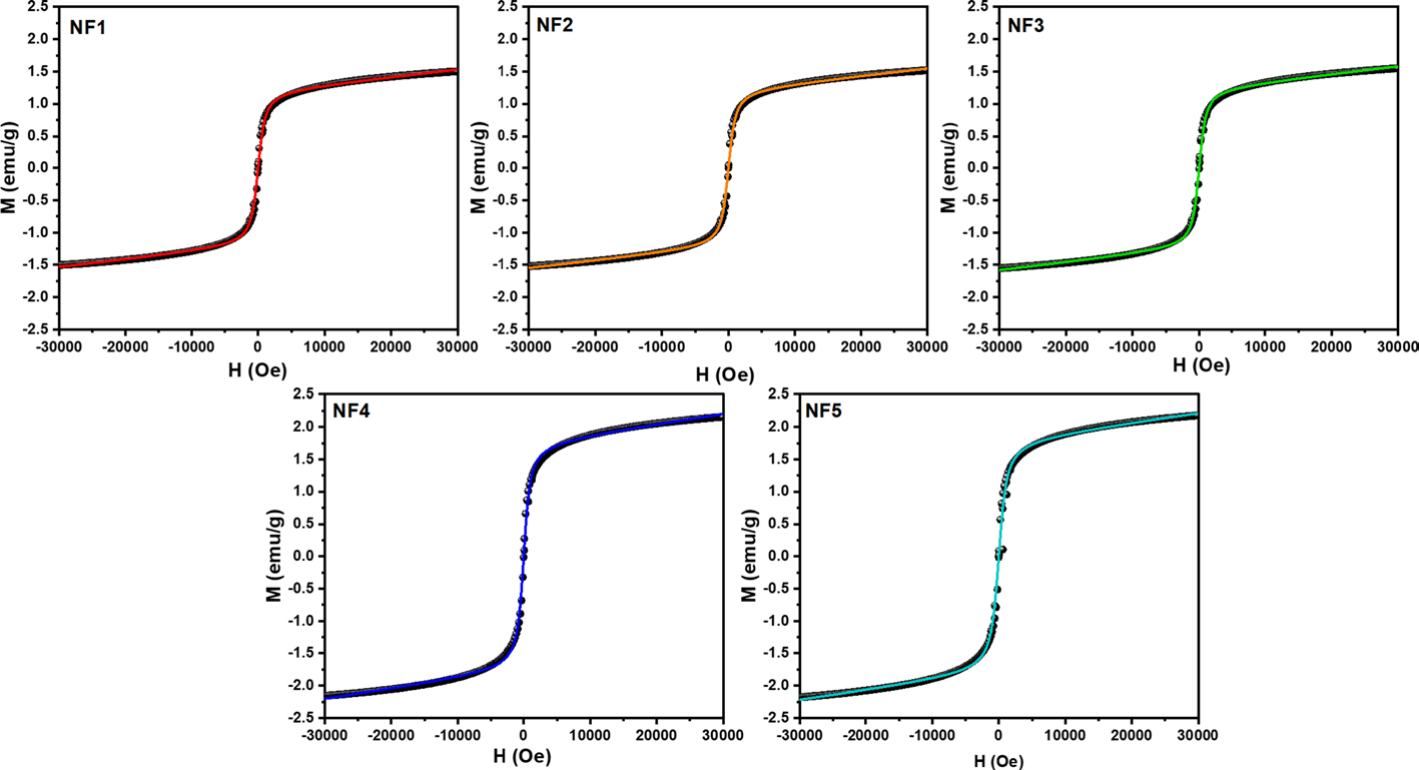
Hysteresis curves of the nanocomposites

The magnetic properties of the zinc oxide/copper ferrite–soursop leaf extract nanocomposites are shown through an “*S*” shaped curve without a hysteresis loop, as presented in Fig. 7. The shape of the curve indicates that the nanocomposites exhibit superparamagnetic properties. This is reinforced by the values of the remanent magnetization (*Mr*) and coercivity field (*Hc*) approaching zero in the range of 0.016 ± 0.009–0.153 ± 0.002 emu/g and (15.86 ± 0.05) × 10^–4^–(563.50 ± 0.05) × 10^–4^ T, respectively (Table 2). This statement is supported by previous research, which states that nanoparticles are superparamagnetic if they have characteristics such as an *S*-shaped curve with *Hc* and *Mr* values approaching zero or can be ignored [43]. Furthermore, based on the results of XRD analysis, it is known that the crystallite sizes of copper ferrite–soursop leaf extract are around 11.75–11.91 nm, while the particle size value of the SEM analysis results of the nanocomposite ranges from 52.44 ± 1.26 nm to 22.68 ± 0.52 nm, where these values have a single domain which is classified as superparamagnetic [58].

**Table 2:** Magnetic properties of the nanocomposites

Samples	*M*_*s*_ (emu/g)	*M*_*r*_ (emu/g)	*H*_*c*_ (Oe)	*H*_*c*_ (T)	$\chi$
NF1	1.455 ± 0.003	0.028 ± 0.008	563.50 ± 0.05	(563.50 ± 0.05) × 1 0^–4^	(1.11 ± 0.01) × 1 0^–4^
NF2	1.472 ± 0.003	0.051 ± 0.008	532.10 ± 0.05	(532.10 ± 0.05) × 10^–4^	(1.09 ± 0.01) × 1 0^–4^
NF3	1.500 ± 0.003	0.016 ± 0.009	204.30 ± 0.05	(204.30 ± 0.05) × 10^–4^	(1.16 ± 0.01) × 1 0^–4^
NF4	2.090 ± 0.003	0.085 ± 0.001	23.51 ± 0.05	(23.51 ± 0.05) × 10^–4^	(1.51 ± 0.02) × 1 0^–4^
NF5	2.130 ± 0.004	0.153 ± 0.002	15.86 ± 0.05	(15.86 ± 0.05) × 10^–4^	(1.39 ± 0.02) × 1 0^–4^

Interestingly, if compared to the bulk form of magnetite, the saturation magnetization (*Ms*) of this research is relatively small, ranging from 1.445 emu/g to 2.130 emu/g. This condition is likely due to the doping of magnetite with copper to form a copper ferrite system and the addition of soursop leaf extract. Furthermore, the addition of non-magnetic materials such as zinc oxide is believed to contribute to the decrease in the magnetization. This condition is similar to previous research, where zinc oxide material was added to copper ferrite, and the *Ms* decreased [38]. Based on Table 2, it is known that the saturation magnetization value increases with the addition of the mass of copper ferrite–soursop leaf extract in the zinc oxide/copper ferrite–soursop leaf extract nanocomposites.

### Antifungal properties of zinc oxide/copper ferrite–soursop leaf extract ferrofluids

**Fig. 8:**
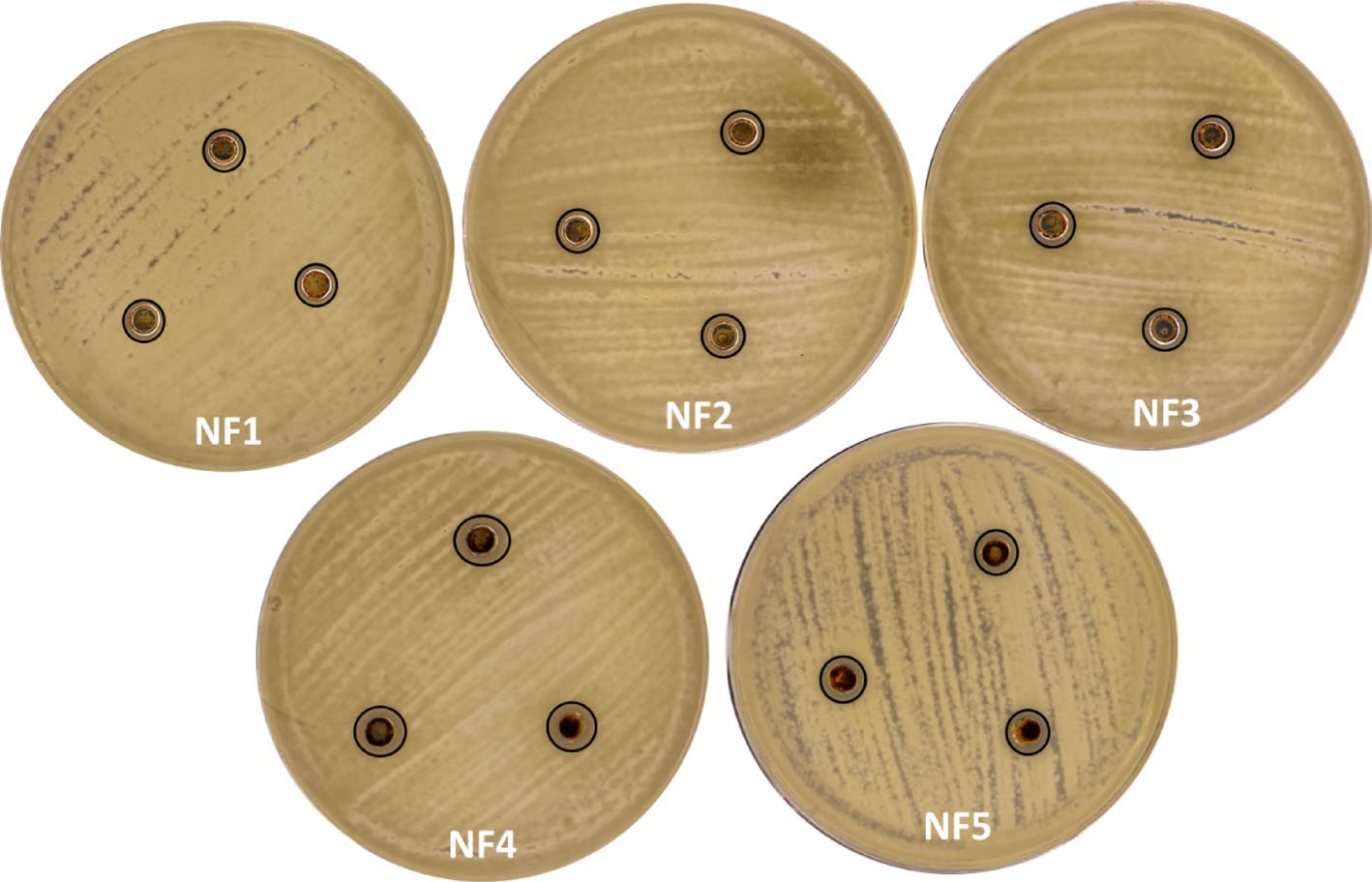
Inhibition zone diameters of the ferrofluids against *A. flavus*

**Fig. 9:**
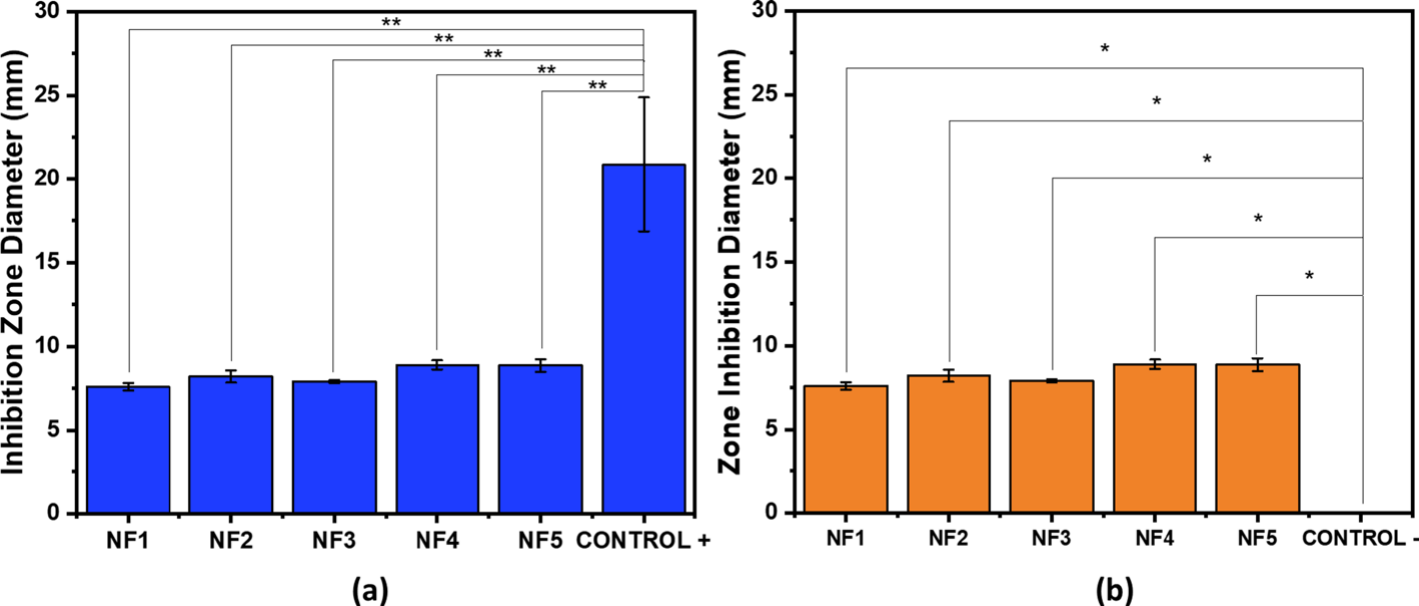
Significant inhibition zone diameter for *p < 0.05

The antifungal activity of zinc oxide/copper ferrite–soursop leaf extract against *A. flavus* is depicted in Fig. 8. The antifungal activity of ferrofluids is reflected by the length of the area not covered by fungi, which is then referred to as the *A. flavus* fungal growth inhibition zone. This zone was then measured horizontally and vertically. The results of the analysis of the measurement of the fungal growth inhibition zone with 3 replicates and standard deviation are presented in Table 3. The average inhibition zone diameter of the zinc oxide/copper ferrite–soursop leaf extract ferrofluids increased slightly with higher nanocomposite content, ranging from 7.60 ± 0.22 mm to 8.90 ± 0.27 mm. In addition to antifungal testing on prepared ferrofluids, tests were also conducted on coconut oil (negative control) as a dispersing medium and surfactant to ensure that the inhibition zone was not caused by these substances. Ketoconazole was used as a positive control for comparison. Table 3 shows that the negative control did not show an inhibition zone, indicating that the resulting inhibition zone originated from the nanocomposites. For the positive control, the diameter of the inhibition zone was approximately 20.87 mm, higher than that of the experimental samples. Nevertheless, the material synthesized in this experiment has promising potential, as its antifungal activity originates from the interaction of nanoparticles with cells in the ferrofluids, which can be further improved through optimization of the synthesis parameters. Furthermore, highlighting their potential for future antifungal applications, the development of ferrofluids is crucial due to their advantages over conventional formulations such as creams, powders, or gels. These advantages relate to the improved dispersion stability and magnetic responsiveness of ferrofluids, allowing for more uniform distribution, including the potential for better targeted delivery. Furthermore, the statistical analysis results shown in Fig. 9 indicate that increasing the mass of copper ferrite–soursop leaf extract had a significant effect (*p < 0.05) on antifungal activity. These results also confirm that the increase in antifungal activity stems from the effectiveness of the ferrofluid system and not from random variation.

**Table 3:** Inhibition zone diameters of the ferrofluids

Samples	Inhibition zone diameters (mm)			
	*Aspergilus Flavus*			
	Zone 1	Zone 2	Zone 3	Average
NF1	7.76	7.35	7.69	7.60 ± 0.22
NF2	8.56	7.83	8.25	8.21 ± 0.37
NF3	7.83	7.91	8.01	7.92 ± 0.09
NF4	9.14	8.60	8.95	8.90 ± 0.27
NF5	8.60	9.30	8.70	8.87 ± 0.38
Negative control (Coconut oil)	0.00	0.00	0.00	0.00
Positive control (Ketoconazole 2%)	23.45	22.90	16.25	20.87 ± 4.01

## Discussion

This study synthesized zinc oxide/copper ferrite–soursop leaf extract ferrofluids. The formation of ferrofluids was confirmed by XRD, FTIR, SEM, and EDX characterizations to determine their crystal structure, functional groups, morphology, and elemental content, respectively. The results of the XRD experiment showed that the diffraction peaks of the samples are consistent with the database for zinc oxide and copper ferrite. Interestingly, no new diffraction peaks other than the copper ferrite peak were detected, despite the presence of soursop leaf extract during synthesis. This suggests that the extract is predicted to act as a surfactant during copper ferrite synthesis [34]. Furthermore, the diffraction pattern did not detect any peak shift in copper ferrite or zinc oxide with increasing mass of copper ferrite-soursop leaf extract. This indicates that adding more copper ferrite does not alter the crystal structure of the nanocomposites. The XRD analysis results also confirmed an insignificant increase in crystallite size of zinc oxide (29.79–29.84 nm) and copper ferrite (11.75–11.91 nm). Increasing the mass of copper ferrite–soursop leaf extract increases the peak intensity, which results in a narrowing of the peak width, resulting in a decrease in the FWHM value. This narrowing causes the nanoparticle crystal size to increase, in accordance with the Debye–Scherrer equation (Eq. 6).

6 $$ D = \frac{K\lambda }{{\beta \cos \theta }} $$

where *D* is the crystal size (nm), *K* is a constant, *λ* is the wavelength value of Cu-Kλ (Å), *β* is the full width at half maximum (rad), and *θ* is the diffraction angle (°). This is also confirmed by previous research, which shows that increasing crystallite size can be associated with narrower peak broadening [59]. The crystallite size of this nanocomposite is smaller compared to previous research, which reported that the size of CuFe_2_O_4_ was 42.30 nm and ZnO was around 35.4–37.8 nm [60, 61]. These results indicate that soursop leaf extract successfully acts as a surfactant.

The FTIR results confirmed the successful synthesis of ferrofluids, indicating the presence of functional groups from fillers, surfactants, and dispersants. The presence of oleic acid is confirmed by the appearance of COO^−^ and CH_2_ vibrations at 1384–1462 cm^−1^ and 2916 cm^−1^ [62, 63]. Meanwhile, the presence of dimethyl sulfoxide as the second surfactant is identified by the appearance of S = O at 953 cm^−1^ [41]. Furthermore, the dispersion in the form of coconut oil is identified in the C–H, C–O, C=O, and unsaturated bonds, which are shown at 1018 cm^−1^, 1313 cm^−1^, 1577–1803 cm^−1^, and 3012 cm^−1^, respectively. The bonds in coconut oil are similar to those in oleic acid. This is because oleic acid is one of the components of coconut oil [64]. Meanwhile, the C–H group indicates the presence of organic compounds from coconut oil [65]. Furthermore, at 400–750 cm^−1^, absorption bands from the metal oxides Fe–O, Cu–O, and Zn–O are detected. The presence of Fe–O at 464 cm^−1^ indicates the presence of vibrations from octahedral Fe–O. In addition to Fe–O octahedral, vibrations from Cu–O also appear in the 400 to 530 cm^−1^ regions. This indicates the success of Cu doping in Fe_3_O_4_ nanoparticles at the octahedral site. Furthermore, the Fe–O tetrahedral vibrations were detected at 580 cm^−1^ and 721 cm^−1^. The analysis confirmed that the copper structure formed is an inverse cubic spinel ferrite [66]. Furthermore, the formation of zinc oxide nanoparticles was identified by Zn–O vibrations at 665 cm^−1^ [67]. In addition to the Zn–O bond, the appearance of the C–H bond at 894 cm^−1^ also confirms the success of the ZnO nanoparticles. These results are consistent with previous research [54]. In addition, the presence of C=O and O=C=O vibrations was detected at 1577–1803 cm^−1^ and 2351 cm^−1^ [68, 69]. The presence of the C=O functional group can be associated with the presence of flavonoids and other polyphenolic compounds found in plant extracts [70]. Furthermore, the C–H stretching vibration detected at 2858 cm^−1^ originates from the methylene and methyl groups, confirming the presence of carbon contained in the plant extracts [71, 72]. The presence of these functional groups confirms the presence of soursop leaf extract, which acts as a surfactant. Both functional groups act as surfactants by attaching to the surface of copper ferrite in the form of a barrier, thereby preventing the surfaces between particles from sticking to each other [34]. In addition to being a surfactant, the content in this extract can also act as an antifungal agent, as reported in previous studies [36, 37]. The intensity of the C–H functional group bond increases along with the increase in the mass of the copper ferrite–soursop leaf extract, reflecting the higher content of the extract in the nanocomposite. The existence of these bonds indicates that zinc oxide/copper ferrite–soursop leaf extract was successfully incorporated into the ferrofluids and functioned effectively as a filler. Additionally, absorption bands observed at 3405 and 3398 cm^−1^ correspond to the presence of O–H vibrations [73, 74], which also increased as the mass of copper ferrite–soursop leaf extract increased. This indicates that the number of hydroxyl groups in the ferrofluids increases, which is certainly beneficial for the formation of free radicals or ROS when applied as an antifungal.

Furthermore, the surface morphology of the nanoparticles and the chemical composition of the nanocomposite surface were confirmed by SEM–EDX results. Based on the results of SEM data analysis, as the mass of copper ferrite-soursop leaf extract increases, the morphology tends to become more uniform, accompanied by reduced agglomeration. The observed reduction in agglomeration is likely due to the increased phytochemical content of soursop leaf extract, which stabilizes the nanoparticles within the nanocomposites [33]. Related to this, previous research also stated that increasing the extract content results in increasingly uniform particle morphology. This mechanism occurs when there is a strong steric interaction between Fe^3+^ ions and bioactive phytochemicals from the extract, causing the particles not to stick to each other [75]. The measurement results obtained smaller particle sizes as the mass of copper ferrite-soursop leaf extract increases (from 52.44 ± 1.26 nm to 22.68 ± 0.52 nm). In this experiment, the particle sizes are larger than the crystallite size shown in the XRD analysis results (Table 1). This occurs because XRD characterizes the size of the crystal domain, while SEM works based on surface morphology, so that the analysis was carried out by measuring the overall particle size, including agglomeration and surface layers. The results of EDX data analysis also confirmed the presence of chemical elements of the nanocomposite O, Fe, Cu, and Zn, which are related to the presence of copper ferrite and zinc oxide particles. In addition, C content was also detected originating from soursop leaf extract added during the synthesis of copper ferrite [76]. Several elements with low composition were detected as impurities, such as Al, Si, Ti, and Cl, predicted to originate from iron sand as a precursor for copper ferrite synthesis [77]. Interestingly, along with the increase in the mass of copper ferrite–soursop leaf extract, an increase in the intensity of the Fe and C spectra was observed, which are the main elements of copper ferrite–soursop leaf extract.

Furthermore, magnetic properties were characterized using VSM. Interestingly, the experimental results showed that all samples exhibited superparamagnetic properties. It was shown that, in addition to the hysteresis curve forming the letter S, the *Hc* and *Mr* values were also close to zero as criteria for particles with superparamagnetic properties [78, 79]. Related to this, previous studies showed that nanoparticles with superparamagnetic properties had *Hc* and *Mr* values of 0.003564 T and 0.00879 emu/g, respectively [80]. Theoretically, superparamagnetic properties occur in nanoparticles with a single domain, where each magnetic domain can have a size of less than 50 nm, but this size depends on the material [79]. In this research, it was found that the nanocomposites had particle sizes in the range of 22.68 nm to 52.44 nm, which are in the range of particles in the superparamagnetic category. The superparamagnetic properties of the nanocomposites contribute to the stability of the ferrofluids and their resistance to agglomeration. This occurs because *Mr* is small, even approaching zero, so when the external magnetic field is removed, the materials will not stick together or be attracted to each other [44]. At this size, nanoparticles can easily interact with fungi, making it easier for nanoparticles to penetrate the fungal membrane and damage important organs [81]. Furthermore, with superparamagnetic properties, it also has advantages in antifungal applications that utilize external magnetic fields as a stimulus so that particles can be properly controlled [78, 79].

The saturation magnetization (*Ms*) values ranged from 1.445 emu/g to 2.130 emu/g, which are relatively low compared to those of Fe_3_O_4_, reported at 58.8 emu/g [73]. Several factors contribute to this low *Ms* value, including the substitution of Cu^2+^ for Fe^2+^ at tetrahedral sites [82–84]. Theoretically, the magnetic moment of Cu^2+^ (1 μB) is lower than that of Fe^2+^ (4 μB), so the total magnetic moment per molecule of copper ferrite is also lower compared to Fe_3_O_4_ (4 μB /molecule), which is 2.5 μB /molecule, calculated using Eq. (7). This causes the saturation magnetization value of copper ferrite to be lower than that of Fe_3_O_4_. Furthermore, the *Ms* value of the nanocomposites is also lower compared to the *Ms* value of copper ferrite from previous research, which reached 53.1 emu/g [73]. The decrease in the *Ms* value is due to the presence of organic materials, such as soursop leaf extract and zinc oxide, which are diamagnetic, and also reduces the magnetization value of the nanocomposites [85, 86]. Interestingly, Table 2 shows that the *Ms* value increases with the incorporation of the copper ferrite–soursop leaf extract. The increase in *Ms* is attributed to the higher content of the magnetic phase in the nanocomposites [87, 88]*.* Moreover, the rise in magnetization values can also be associated with an increase in primary particle or crystallite sizes (Table 1). Larger particles exhibit greater magnetic domain alignment, leading to higher saturation magnetization. Meanwhile, for smaller particles on the nanometer scale, there is a misalignment of the magnetic domains on a larger specific surface area, leading to a decrease in *Ms* value [89].

7 $$ \left( {{\mathrm{Fe}}^{3 + } } \right)_{{{\mathrm{tetrahedral}}}} \left[ {{\mathrm{Fe}}_{1}^{3 + } {\mathrm{Fe}}_{{1 - {\mathrm{x}}}}^{2 + } {\mathrm{Cu}}_{{\mathrm{x}}}^{2 + } } \right]_{{{\mathrm{octahedral}}}} {\mathrm{O}}_{4} $$

To determine the antifungal activities, zinc oxide/copper ferrite-soursop leaf extract ferrofluids were tested using the well method, and the inhibition zone diameter increased with increasing mass of zinc oxide/copper ferrite-soursop leaf extract from 7.60 ± 0.22 mm to 8.90 ± 0.27 mm. These results indicate that ferrofluids can inhibit the growth of *A. flavus* in the resistant category, as the inhibition zone obtained was less than 14 mm [90]. The increase in the inhibition zone diameter likely occurs because the smaller particle size provides a larger specific surface area for interaction with pathogens and allows it to penetrate the fungal cell membrane more easily, thereby increasing antifungal activity [28, 91]. Based on the SEM images (Fig. 4), it is known that the agglomeration of the nanocomposites decreases with increasing mass of copper ferrite-soursop leaf extract. This decrease in agglomeration corresponds to a decrease in particle size from 52.44 ± 1.26 nm to 22.68 ± 0.52 nm, which conceptually increases the specific surface area of the particles interacting with the fungus. With their smaller size, nanoparticles more easily penetrate and damage fungal membranes and cells [92]. In addition, the increase in the inhibition zone diameter can also be attributed to the presence of more O–H functional groups with increasing mass of copper ferrite-soursop leaf extract, as confirmed by the FTIR spectrum (Fig. 3). These O–H functional groups also contribute to the formation of oxidative stress (ROS), where ROS induces oxidative stress that results in fungal cell damage, thus inhibiting fungal growth [93, 94]. Furthermore, oxidative stress caused by ROS will damage proteins, lipids, and fungal DNA, resulting in changes in biological activity, accelerated mutations, and cell death [95].

Interestingly, when compared to the inhibition zone diameters of zinc oxide and copper ferrite reported by previous studies, which reached 2–8 mm and 6 mm, respectively [96, 97], the ferrofluids in this work showed larger values, indicating stronger antifungal activity. This increase in the inhibition zone diameter can be attributed to the modification in the ferrofluids with the use of dual surfactants, namely dimethyl sulfoxide and oleic acid. Dimethyl sulfoxide is known to increase fungal membrane permeability, thereby facilitating the penetration of oleic acid into fungal cells, which subsequently destabilizes fungal lipid membranes and enhances the antifungal effect of ferrofluid [41, 47, 48]. Other factors that increase inhibition zone diameter may be associated with the presence of bioactive compounds, such as alkaloids, flavonoids, phenolics, and saponins, originating from soursop leaf extract [98]. Previous research showed that bioactive compounds can inhibit vital fungal enzymes in DNA replication and reproduction, are able to form complexes with proteins, damage membranes, and destroy cytoplasm [99, 100]. Therefore, these bioactive compounds play a significant role in inhibiting fungal growth.

**Fig. 10:**
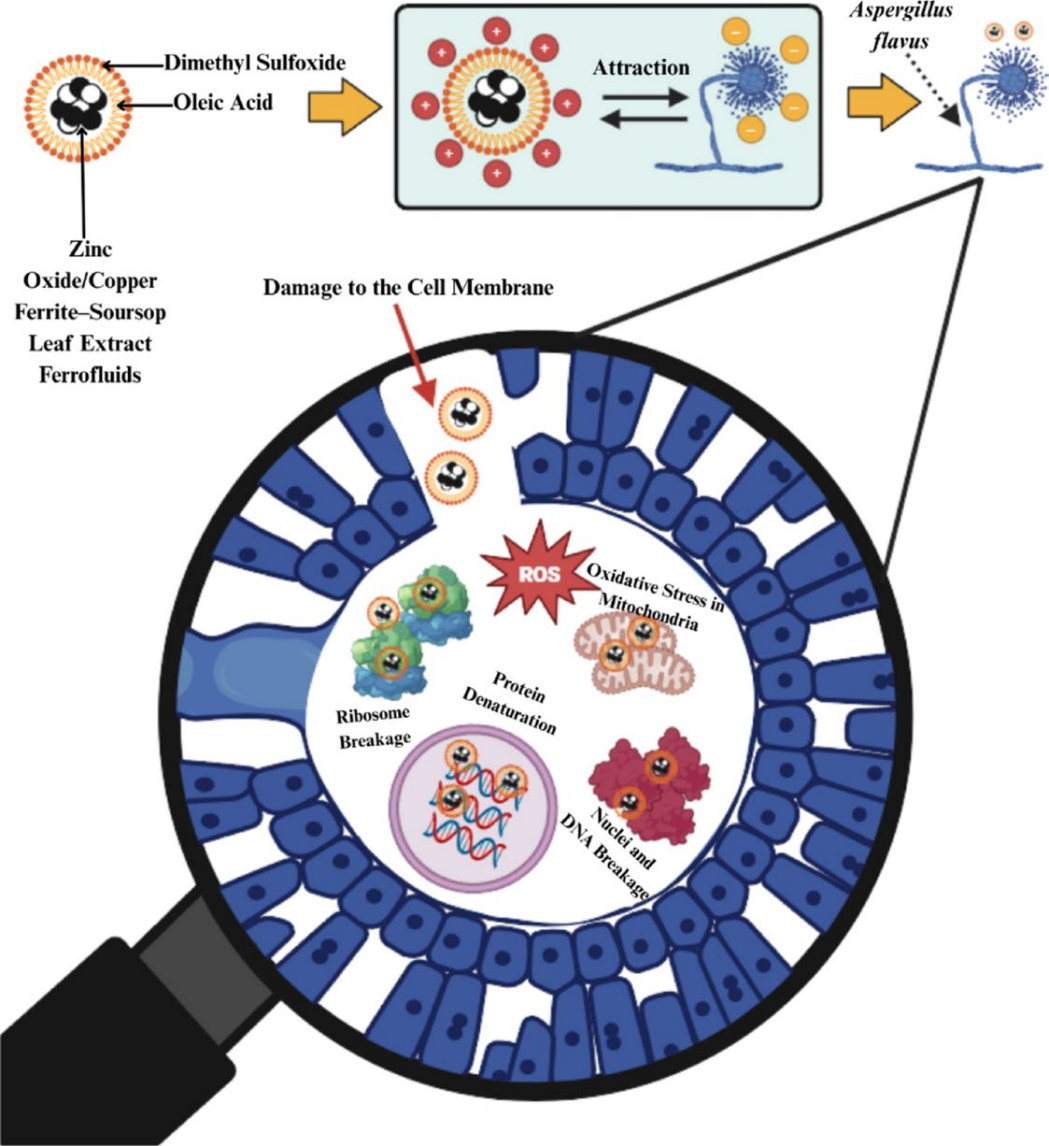
Proposed antifungal mechanism of the zinc oxide/copper ferrite–soursop leaf extract ferrofluids

The proposed action mechanism of the zinc oxide/copper ferrite–soursop leaf extract ferrofluids in inhibiting the growth of *A. flavus* is illustrated in Fig. 10, which can occur when ferrofluids encounter *A. flavus*. First, the ferrofluids composed of positively charged zinc oxide/copper ferrite (Cu^2+^, Fe^3+^, and Zn^2+^) attract the negatively charged fungal surface through electrostatic forces. DMSO, the outermost surfactant, acts as a permeability-enhancing agent because it facilitates pore formation and opens the cell membrane [101]. After the cell membrane opens, oleic acid (as the first surfactant) functions to increase lipid fluidity, disrupt membrane integrity, and disrupt ergosterol biosynthesis so that membrane stability decreases [102, 103]. This allows ferrofluids to penetrate fungal cell membranes, leading to interactions between the ferrofluids and the fungi, potentially triggering the formation of reactive oxygen species, including peroxide radicals (O_2_^−^), hydroxyl radicals (OH^−^), hydrogen peroxide (H_2_O_2_), and singlet oxygen (^1^O_2_), which effectively inhibit fungal growth [67]. Finally, ROS penetrates the fungal cell through pores and damages nuclear DNA, proteins, mitochondria, and ribosomes, thus causing fungal cell death [104, 105].

## Conclusions

Ferrofluids with zinc oxide/copper ferrite–soursop leaf extract filler as antifungal agents for *A. flavus* derived from natural materials in the form of iron sand, soursop leaves, and coconut oil were successfully fabricated using double surfactants. The formation of the nanocomposite as a ferrofluid filler was confirmed through XRD, FTIR, and SEM analysis. Copper ferrite nanoparticles showed an inverse cubic spinel structure with Cu–O and Fe–O bonds in octahedral positions and Fe–O in tetrahedral positions, while zinc oxide showed a hexagonal wurtzite structure. The presence of soursop leaf extract was detected from C=O, C–H, and O–H. Meanwhile, the presence of OA and DMSO, as well as coconut oil as surfactants and dispersants, is proven by the appearance of functional groups COO^−^, CH_2_, S=O, C–H, C–O, C=O, and unsaturated bonds. The nanocomposites exhibited a tendency toward spherical shapes and greater homogeneity with decreasing particle sizes as the copper ferrite-soursop leaf extract content increased, from 52.44 ± 1.26 nm to 22.68 ± 0.52 nm. Zinc oxide/copper ferrite–soursop leaf extract ferrofluids exhibited antifungal activity against *A. flavus* with an inhibition zone diameter increasing from 7.60 ± 0.22 mm to 8.90 ± 0.27 mm. This increase is associated with decreased agglomeration and particle size, thus increasing the surface area interacting with the fungus. In addition, the increase in O–H functional groups, confirmed by FTIR results, also influenced the formation of ROS. The presence of bioactive compounds was also a factor in the performance of ferrofluids in inhibiting the growth of *A. flavus*. Although the inhibition zone diameter in this study is still in the resistant category with a lower value when compared to ketozonacole, the study of zinc oxide/copper ferrite–soursop leaf extract ferrofluids as antifungal agents, especially for *A. flavus*, using natural ingredients, is a novelty that is expected to be an alternative in the treatment of fungal infections in the future. Therefore, further investigation is essential by modifying surface functionalization to improve antifungal selectivity in the future, including comprehensive biocompatibility and toxicity evaluations.

## Data Availability

The datasets generated during and/or analysed during the current study are available from the corresponding author on reasonable request.
